# Quantitative analysis of organelle distribution and dynamics in *Physcomitrella patens* protonemal cells

**DOI:** 10.1186/1471-2229-12-70

**Published:** 2012-05-17

**Authors:** Fabienne Furt, Kyle Lemoi, Erkan Tüzel, Luis Vidali

**Affiliations:** 1 Department of Biology and Biotechnology, Worcester Polytechnic Institute, 100 Institute Road, Worcester, MA, 01609, USA; 2 Department of Physics, Worcester Polytechnic Institute, 100 Institute Road, Worcester, MA, 01609, USA

**Keywords:** Organelle distribution, organelle dynamics, tip growth, *Physcomitrella patens*

## Abstract

**Background:**

In the last decade, the moss *Physcomitrella patens* has emerged as a powerful plant model system, amenable for genetic manipulations not possible in any other plant. This moss is particularly well suited for plant polarized cell growth studies, as in its protonemal phase, expansion is restricted to the tip of its cells. Based on pollen tube and root hair studies, it is well known that tip growth requires active secretion and high polarization of the cellular components. However, such information is still missing in *Physcomitrella patens*. To gain insight into the mechanisms underlying the participation of organelle organization in tip growth, it is essential to determine the distribution and the dynamics of the organelles in moss cells.

**Results:**

We used fluorescent protein fusions to visualize and track Golgi dictyosomes, mitochondria, and peroxisomes in live protonemal cells. We also visualized and tracked chloroplasts based on chlorophyll auto-fluorescence. We showed that in protonemata all four organelles are distributed in a gradient from the tip of the apical cell to the base of the sub-apical cell. For example, the density of Golgi dictyosomes is 4.7 and 3.4 times higher at the tip than at the base in caulonemata and chloronemata respectively. While Golgi stacks are concentrated at the extreme tip of the caulonemata, chloroplasts and peroxisomes are totally excluded. Interestingly, caulonemata, which grow faster than chloronemata, also contain significantly more Golgi dictyosomes and fewer chloroplasts than chloronemata. Moreover, the motility analysis revealed that organelles in protonemata move with low persistency and average instantaneous speeds ranging from 29 to 75 nm/s, which are at least three orders of magnitude slower than those of pollen tube or root hair organelles.

**Conclusions:**

To our knowledge, this study reports the first quantitative analysis of organelles in *Physcomitrella patens* and will make possible comparisons of the distribution and dynamics of organelles from different tip growing plant cells, thus enhancing our understanding of the mechanisms of plant polarized cell growth.

## Background

Plant cells display a large variety of shapes and morphologies, such as the puzzle-like leaf epidermal cells and the filament-like elongated root hairs. Such diversity is made possible by the mechanism of differential cell growth, in which cell wall and plasma membrane extension occur at a confined region, and which requires a dynamic coordination of the cytoskeleton and the endomembrane system [1-3]. For example, in pollen tubes and root hairs which undergo polarized cell growth, expansion is restricted to the tip of the elongating cells. The moss *Physcomitrella patens* is a particularly suitable plant model system to investigate polarized cell growth, not only for its amenability to genetic studies [4-6], but also because during its haploid gametophyte phase, its protonemata expand by a specific type of polarized growth called "tip growth" [7]. Moss protonemata are composed of two types of elongated cells: chloronemata which are highly vacuolated, contain large chloroplasts and are mainly involved in photosynthesis, and caulonemata which are thinner, longer, contain a basal vacuole and fewer chloroplasts, and are implicated in land colonization and nutrient acquisition [8,9]. Ultrastructure analyses conducted on different moss species, such as *Funaria hygrometricaPhyscomitrium turbinatum*, dark-grown *Ceratodon purpureus* and *Physcomitrella patens*, showed that chloronemata have a homogeneous organelle distribution while caulonemata have a differentiated cytoplasmic organization [10-13]. In a recent study, the morphology of the vacuole in *Physcomitrella patens* chloronemata was investigated in more details using a GFP fusion of a t-SNARE as a tonoplast marker [14]. It was shown that vacuoles in apical chloronemata are highly dynamic and complex with tubular protrusions while their structure is much simpler in sub-apical cells. However, our current knowledge on the distribution and dynamics of the organelles in *Physcomitrella* remains very limited and further analyses are needed to assess the participation of organelle organization in tip growth, and how this organization is achieved and maintained in moss protonemata.

Numerous studies have reported that pollen tubes and root hairs, two tip growing plant cells, display a structural and functional compartmentalization of their cytoplasm and their organelles, which is thought to be essential for tip growth [15-19]. Early works reported a “zonation” of the cytoplasm in pollen tubes from angiosperms but the chemical fixation traditionally employed for these ultrastructure studies was found to affect the distribution of organelles [16]. In the 1990s, the application of rapid freeze fixation and freeze substitution techniques resulted in a more representative picture of the *in vivo* cytoplasmic organization [20-22]. The extreme apex of the pollen tube tip consists almost exclusively of highly motile vesicles often referred to as an inverted cone-shaped domain. The large organelles are distributed throughout the rest of the cell with mitochondria and tubular ER particularly concentrated in a “clear zone” flanking the vesicle-rich region and the Golgi dictyosomes in the sub-apical region. Plastids and large vacuoles mostly occupy the distal region behind the clear zone. Derksen et al. (1995) also performed a morphometric and quantitative analysis of some organelles in tobacco pollen tubes [21]. They showed that mitochondria form round or oval structures up to 2.5 μm in length and that Golgi dictyosomes are homogeneous in size (< 1 μm) and shape, and contain 4 to 6 cisternae. Both organelles are mostly oriented parallel to the longitudinal axis of the tube in the shank, but exhibit different orientations where the cytoplasmic streaming changes direction. The first 40 μm of tobacco pollen tubes were estimated to contain ~130 mitochondria and ~ 40 Golgi dictyosomes [21]. A similar polarized distribution of the cytoplasm and the organelles has been described in growing root hairs from different species, such as Arabidopsis, vetch or *Medicago truncatula*[15,23-27]. The apical zone, which is also packed with vesicles, is slightly smaller than in pollen tubes, less than 10 μm from the apex, followed by a ~ 40 μm organelle-rich sub-apical region and a vacuolated basal region.

In the late 1990s, advances in molecular biology and development of fluorescent protein fusions with specific organelle enzymes or resident proteins allowed for the visualization of single organelles in live cells [28]. A set of fluorescent organelle markers is now available in plants, a result of computational approaches to identify targeting motifs for each organelle [29-31]. Using these techniques, several independent groups have confirmed the observations from ultrastructure studies of the distribution of organelles in pollen tubes and root hairs. In both lily and tobacco, the GFP fused to the ER retention motif HDEL labels a complex interconnected network, present throughout the pollen tube with enhanced density behind the inverted cone [19,22,32]. Tobacco pollen tubes expressing a GFP fusion protein targeting the mitochondria show the same distribution as the ER marker and label individual, short filament-like structures, while lily pollen tubes and Arabidopsis root hairs mitochondria traced with the fluorescent probe MitoTracker display a branched network of less defined structures [19,22,32,33]. A set of GFP fusions with different Rab proteins was used to specifically visualize Golgi dictyosomes which exhibit a punctated pattern throughout the tobacco pollen tube except at the inverted cone, as well as endosomes and secretory vesicles which accumulate at the tip [19,32]. The Golgi dictyosomes distribution was also investigated in Arabidopsis root hairs using a different fluorescent protein, the resident enzyme sialyl transferase, and proved to be similar to that of pollen tubes [34]. The peroxisome-targeted GFP labeled discrete, punctuted structures in tobacco pollen tube and was absent from the clear zone [19,32]. The expression of fluorescent markers has also proved to be a more appropriate and powerful tool to investigate organelle dynamics in live cells. For example, organelles in pollen tubes from tobacco were shown to move with instantaneous velocities ranging from 0.5 to 1.5 μm/s [35,36]. The instantaneous velocity of Golgi dictyosomes, mitochondria and peroxisomes measured in Arabidopsis root hairs was ~ 1 μm/s [34,37].

In this article, we provide the first quantitative analysis of organelles in *Physcomitrella patens* using live cell microscopy. We found that the distribution of the organelles in protonemata follows a tip-high gradient, and that these organelles move with low persistency and average instantaneous speeds in the nm/s range. By comparing the distribution and dynamics of organelles from different tip growing plant cells, we aim to better understand the importance of organelle organization for plant tip growth.

## Results

### Experimental design and analysis

**Figure 1:**
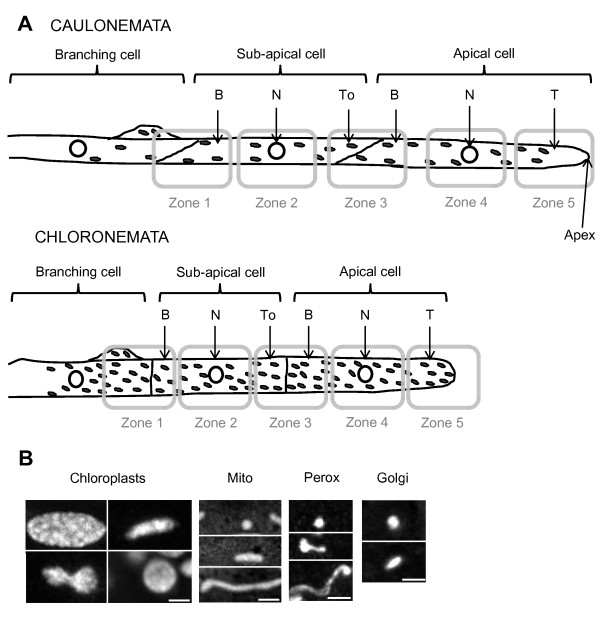
** Experimental design of organelle imaging in*Physcomitrella patens*protonemata.** (**A**) Five zones have been imaged along caulonemata and chloronemata and six areas have been defined for the quantitative analysis with B: Base; N: Nuclear area; To: Tip-oriented area; T: Tip. (**B**) Representative morphologies of chloroplasts, mitochondria (Mito), Golgi dictyosomes (Golgi) and peroxisomes (Perox) detected in moss protonemal cells. Scale bars 2 μm.

To determine the distribution and the dynamics of the organelles in live moss protonemal cells, we used fluorescent protein fusions to visualize and track Golgi dictyosomes, mitochondria, and peroxisomes, and chlorophyll auto-fluorescence for chloroplasts. To assure the reproducibility of our measurements, we chose to image one week-old moss filaments in which the third cell from the tip had started branching. Five zones centered either on the tip, the nuclear region or the cell wall region were defined along the apical and sub-apical protonemal cells expressing a fluorescent organelle marker as shown in figure 1A. It is important to note that each zone was 43 to 48 μm in length and that the average total length of the apical and sub-apical cells was 157.2+/−34.7 μm and 116+/−11 μm for caulonemata, and 102.3+/−18.7 μm and 75.6+/−10.9 μm for chloronemata, showing that the whole cells (or the majority in the case of apical caulonemal cells) were imaged. However, as the cell wall zones (3 and 1) include two cells, we split these zones into two different areas for the quantitative analysis as shown in figure 1A. We first performed a morphometric analysis of all organelles studied; figure 1B summarizes the different organelle morphologies encountered in moss cells. For the quantitative analysis, after confocal imaging, the volume of each entire zone was calculated, and after image processing and analysis in ImageJ, the number of organelles detected in each zone was determined as described in Methods. From these data, the organelle density was calculated in each zone. After defining the average volume of each cell and the average density per cell of each organelle, we extrapolated the total number of organelles present in each cell.

### Chloronemata contain significantly more chloroplasts than caulonemata

**Figure 2:**
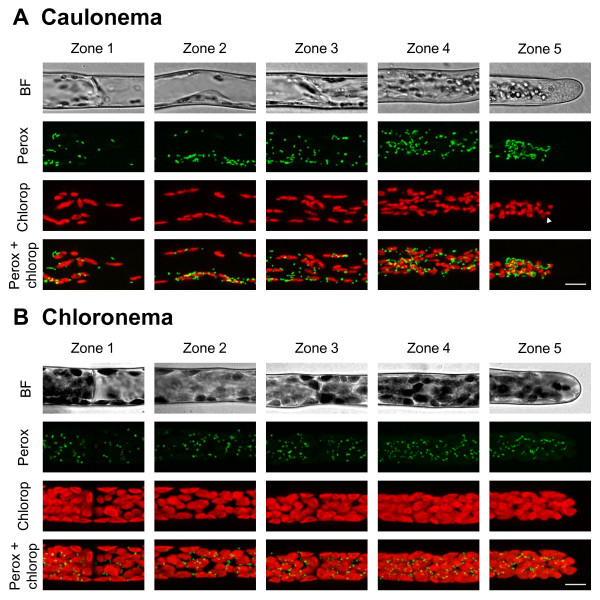
** Peroxisomes and chloroplasts distribution in*Physcomitrella patens*protonemata.** Bright field and confocal images of 5 distinct zones in caulonemata (**A**) and chloronemata (**B**) expressing the CFP-SKL fusion protein to visualize peroxisomes (Perox). The chloroplasts (Chlorop) are visualized by chlorophyll autofluorescence. The top panel shows the bright field (BF). Green and red signals represent CFP and chlorophyll autofluorescence respectively. Bottom panels show the merged image of the green and red signals. Images are displayed as maximal projections of confocal sections. Arrowhead shows dividing chloroplasts. Scale bar 10 μm.

**Figure 3:**
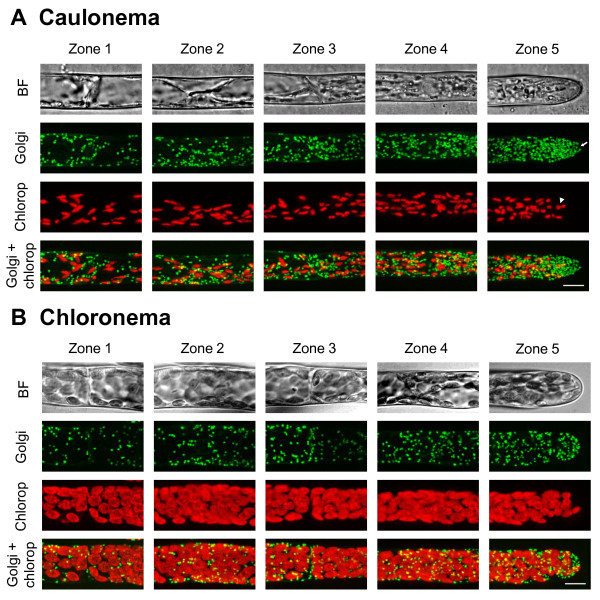
**Golgi dictyosomes and chloroplasts distribution in*Physcomitrella patens*protonemata.** Bright field and confocal images of 5 distinct zones in caulonemata (**A**) and chloronemata (**B**) expressing the YFP-Man fusion protein to visualize Golgi dictyosomes (Golgi). The chloroplasts (Chlorop) are visualized by chlorophyll autofluorescence. The top panel shows the bright field (BF). Green and red signals represent YFP and chlorophyll autofluorescence respectively. Bottom panels show the merged image of the green and red signals. Images are displayed as maximal projections of confocal sections. Arrow and arrowhead show respectively small area deprived of Golgi dictyosomes and dividing chloroplasts. Scale bar 10 μm.

**Figure 4:**
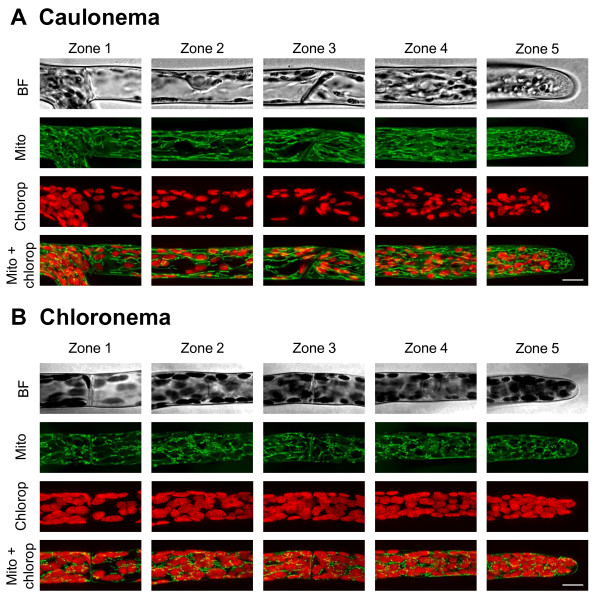
** Mitochondria and chloroplasts distribution in*Physcomitrella patens*protonemata.** Bright field and confocal images of 5 distinct zones in caulonemata (**A**) and chloronemata (**B**) expressing the mEGFP-Cox fusion protein to visualize mitochondria (Mito). The chloroplasts (Chlorop) are visualized by chlorophyll autofluorescence. The top panel shows the bright field (BF). Green and red signals represent mEGFP and chlorophyll autofluorescence respectively. Images are displayed as maximal projections of confocal sections. Scale bar 10 μm.

**Figure 5:**
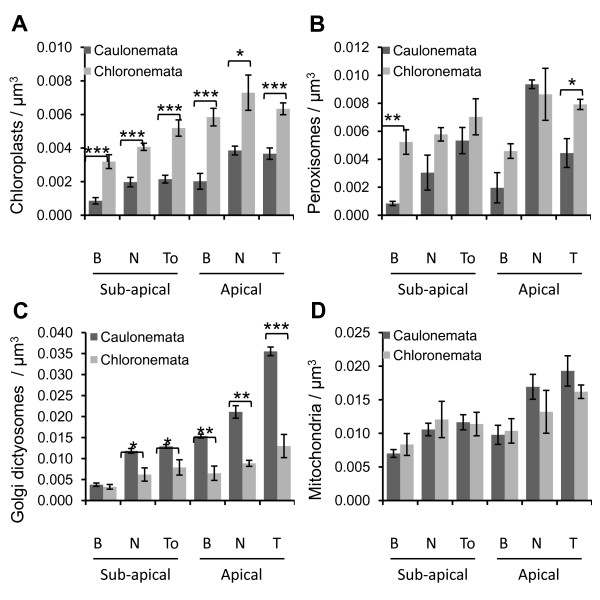
** Organelles density in*Physcomitrella patens*protonemata.** Density of chloroplasts (**A**), peroxisomes (**B**), Golgi dictyosomes (**C**) and mitochondria (**D**) detected in 6 distinct zones of caulonemata and chloronemata with B: Base; N: Nuclear area; To: Tip-oriented area; T: Tip. Results are expressed as the mean value of the number of cells analyzed (chloroplasts, *n* = 9; peroxisomes, *n* = 3; Golgi dictyosomes, *n* = 4 for caulonema and *n* = 3 for chloronema; Mitochondria, *n* = 6 for caulonema and *n* = 3 for chloronema). Error bars indicate the standard error of the mean (samples are statistically different with the following error probabilities: * *P* < 0.05; ** *P* < 0.01; *** *P* < 0.001 by *t*-test).

Chloroplasts and plastids in general are large organelles which can easily be observed by light microscopy. Therefore, the difference in chloroplast content is often used as a criterion to distinguish chloroplast-enriched chloronemata from chloroplast-depleted caulonemata. However, quantitative studies of organelles are still needed to fully understand the different functions fulfilled by these two cell types and particularly how they both achieve and maintain polarized tip growth. In order to determine the chloroplast distribution in moss protonemata, chlorophyll, which has the property to fluoresce red when excited by a 488 nm laser, was used as a fluorescent marker. We observed, as other groups did, that chloroplasts in chloronemata display a large ovoid shape (Figures 2B3B4B) [8]. We also determined that they can reach up to 7 μm in length and 4 μm in width. In contrast, the population of chloroplasts in caulonemata exhibits more diversity in terms of shape and size. They are mostly round and small (2–3 μm) in apical cells and more elongated with a length up to 4.5 μm in sub-apical cells (Figures 2A3A4A). Such variation in chloroplast morphology was observed in higher plants depending on the tissues and in other moss species depending on the differentiation stage [8,9,38]. In addition, the distribution of chloroplasts is not homogeneous throughout the caulonemata, for they are absent from the extreme tip of the apical cell, 9 to 15 μm from the apex, and become peripheral in the sub-apical cells due to the voluminous vacuoles as shown in Figures 2A3A4A. Moreover, we determined that each zone in chloronemata contains significantly more chloroplasts than their caulonemata homologs (figure 5A); we estimated that apical and sub-apical cells contain 146+/−36 and 82+/−15 chloroplasts, respectively for chloronemata *versus* 118+/−24 and 52+/−18 for caulonemata (Means ± SD of 9 cells each). Despite these differences, chloroplasts are distributed in a gradient along both filaments, the chloroplast density being significantly higher at the tip of the apical cells than at the base of the sub-apical cells (Figures 2,3,4,5A, See Additional file 1). A gradient was also observed within cells. In apical and sub-apical cells from caulonemata, the tip or tip-oriented area and the nucleus area display a significantly higher amount of chloroplasts than the base area (See Additional file 2). Similarly, in chloronemata, the tip-oriented area contains significantly more chloroplasts than the base of the sub-apical cells (See Additional file 3).

### Peroxisomes display similar distribution to that of chloroplasts in moss protonemata

The peroxisome marker was designed, based on the plant peroxisome targeting signal (PTS) [29,39,40], by fusing the PTS type 1 SKL to the C-terminus of the CFP (CFP-SKL). Both caulonemata and chloronemata contain peroxisomes of various sizes and shapes within a single cell (figure 2). Most of them are spherical with a diameter between 0.4 and 0.6 μm, and tubular extensions of 1–2 μm in length, called peroxules [41], are frequently observed in caulonemata and only occasionally in chloronemata. These results are in agreement with those of Arabidopsis and tobacco leaf epidermal cells, onion epidermal cells and tobacco pollen tubes [19,30,41,42]. However, in the tip area of the caulonemata, peroxisomes mostly appear as twisted filaments which can reach up to 8 μm in length and are excluded from the extreme tip of the cells as previously described for the chloroplasts (figure 2A). We noticed that peroxisomes are often found in close proximity with chloroplasts in both cell types (data not shown). We determined that the extrapolated total number of peroxisomes in chloronemata is 159+/−35 for apical cells and 119+/−26 for sub-apical cells, and in caulonemata, 186+/−45 and 93+/−29 (Means ± SD of 3 cells each; figure 5, See Additional file 4). As for chloroplasts, peroxisomes are distributed in a gradient within cells in caulonemata. There is a significantly higher density in the tip area *versus* the nucleus area and in the nucleus area *versus* the base of the apical cells, and in the tip-oriented area *versus* the base of the sub-apical cells (See Additional file 2). However, one could note that the peroxisome density at the tip is 2.1 times lower than at the nucleus area. This is due to the fact that the region between the tip and the nucleus in the apical caulonemata contains filament-like peroxisomes that are bigger structures than the punctuted ones and counted as a single object. A gradient was also detectable along the filament in chloronemata as the tip of the apical cells exhibits a significantly higher density than the base of the sub-apical cell (See Additional file 1).

### Golgi dictyosomes accumulate at the tip of the caulonemal apical cells

The distribution of the Golgi dictyosomes in *Physcomitrella* protonemata was investigated by expressing the fusion of the coding sequence of YFP to the first 49 amino acids of the soybean α-1,2-mannosidase (YFP-Man), a well-established marker of Golgi dictyosomes for co-localization studies in plants [30,43]. According to their orientation in the cells, dictyosomes appear either as small round discs with a diameter ranging from 0.6 to 1.4 μm or as thin ellipses of about 1–1.5 μm in length (figure 3) which is consistent with the results reported in Arabidopsis leaf epidermal cells and tobacco pollen tubes [21,30,34]. The quantitative analysis revealed that the multiple Golgi dictyosomes are distributed in a dramatic gradient in caulonemata as shown in Figures 3A and 5C. The Golgi dictyosomes density at tip of the apical cells is 1.7 times higher compared to that of the nucleus area of the same cell, and 9.4 times higher compared to that of the base area of the sub-apical cells: these differences are highly significant (figure 5C, See Additional file 5, Additional file 1, Additional file 2). Moreover, at the tip of the apical cells, Golgi dictyosomes were not homogeneously distributed. They particularly accumulated in a region between 2–5 and 10–15 μm from the apex (figure 3A), but strikingly, they were not excluded from the extreme tip of the caulonemata, like chloroplasts and peroxisomes. Occasionally, a small area of 1–2 μm in diameter deprived of Golgi dictyosomes could be seen at the tip (see arrow in figure 3A). A gradient was observed to a lesser extent in chloronemata, with the tip of the apical cells that display a significantly higher Golgi dictyosomes density than that of the base of the sub-apical cells (See Additional file 1). Figures 3 and 5C also demonstrate that caulonemata contain significantly more Golgi dictyosomes than chloronemata. We estimated that apical and sub-apical cells contain respectively 850+/−64 and 289+/−15 (Means ± SD of 4 cells each) Golgi dictyosomes in caulonemata *versus* 213+/−64 and 115+/−44 (Means ± SD of 3 cells each) in chloronemata.

### Mitochondria display homogeneous distribution but distinct morphology in protonemata

The mitochondrial marker was generated by combining the first 29 amino acids of the yeast cytochrome c oxidase IV ScCOX4 and the coding sequence of the mEGFP (mEGFP-Cox) according to Kölher et al. [44] and Nelson et al. [30]. In all six caulonemata selected for this study, the majority of the mitochondria formed sausage-like structures between 5 and 10 μm long and which were oriented parallel to the cell axis (figure 4A). Shorter filament-like structures and punctuted elements with a diameter of 0.5-1.2 μm were also observed in the apical cell (figure 4A). In addition, mitochondria were homogeneously distributed throughout the caulonemata up to 2–3 μm from the apex. In contrast, chloronemata exhibited almost exclusively short sausage-like structures as well as round elements (figure 4B). Such variation in size and shape of the mitochondria was also reported in Arabidopsis epidermal leaves [30] while they appear more homogeneous in tobacco pollen tubes [19,21]. Although both cell types display a distinct mitochondrial network, they display a similar density of elements in each selected area (figure 5D, See Additional file 6). We estimated that caulonemata contain 543+/−44 mitochondria in apical cells and 296+/−56 in sub-apical cells (Means ± SD of 6 cells each) and chloronemata 299+/−60 and 210+/−33 (Means ± SD of 3 cells each), respectively. These results also indicate that similar to the other organelle data, more structures are found in the apical cells as compared to the sub-apical cells. Accordingly, the tip of the apical cells in both filaments contains a significantly higher density than that of the base of the sub-apical cells (See Additional file 1).

### Organelles move slowly and with low persistency in moss protonemata

**Figure 6:**
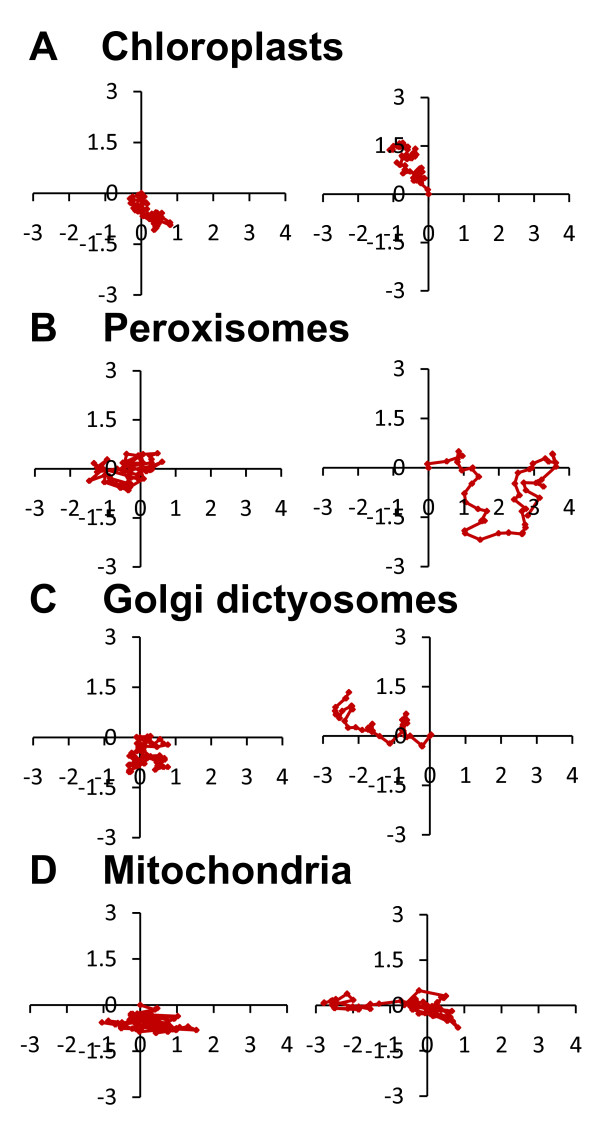
** Organelles motility in tip gowing*Physcomitrella patens*caulonema.** Two representative trajectories of chloroplasts (**A**), peroxisomes (**B**), Golgi dictyosomes (**C**) and mitochondria (**D**) are shown. Trajectories have been built from time lapse series in which images were acquired at 5 s intervals for 5 min. Scale unit: μm.

To understand the dynamics of the organelles in *Physcomitrella* caulonemata, their trajectories were monitored in a time series, every 5 s for 5 min (See Additional file 7Additional file 8Additional file 9Additional file 10), using the fluorescent markers previously described, and the three following parameters were examined: the average instantaneous speed, which corresponds to the distance traveled in 5 s *i.e.* the smallest time interval; the displacement rate, which represents the shortest distance traveled in 5 min *i.e.* between the first and the last time points, and the persistency, which is the ratio average instantaneous speed/displacement rate. As shown in Table 1, chloroplasts which move with an average instantaneous speed of ~ 30 nm/s are the slowest organelles, and mitochondria with ~ 75 nm/s the fastest; the maximum instantaneous speed recorded for the mitochondria was 90.6 nm/s. However, both organelles display a similar displacement rate which means that they travel the same absolute distance in 5 min. Consequently, chloroplasts are more persistent that mitochondria, for they move slowly towards a direction, while mitochondria move mainly around the same position. The most persistent organelles are Golgi dictyosomes. Even though their average instantaneous speed is rather slow at 45 nm/s, they move further than the other organelles during the same amount of time. As previously mentioned, we observed that several peroxisomes are in close proximity to chloroplasts and that both organelles display a similar distribution in moss cells (figure 2), therefore, we expected that they would have similar dynamics. Interestingly, the motility of peroxisomes was distinct from that of chloroplasts; although they move twice as fast, they are not as persistent. In addition, the average instantaneous speeds were relatively homogeneous in the population of tracked organelles from each cell line, while important variations were reported for the displacement rate. The lowest displacement rates measured were 1.2, 1.3 and 2.4 nm/s and the highest were 12.8, 21.2 and 19 nm/s for the mitochondria, Golgi dictyosomes and peroxisomes, respectively. Stop-and-go movements were occasionally observed for Golgi dictyosomes, peroxisomes and mitochondria (See Additional file 8, Additional file 9, Additional file 10). Such diversity is illustrated in figure 6 and see Additional file 11, Additional file 12, Additional file 13, Additional file 14, which display the trajectories of all organelles tracked for this study, and is consistent with the data describing the organelle trajectories in tobacco pollen tube [35].

**Table 1:** Organelle speeds and persistency in tip growing *Physcomitrella patens* caulonema

	Average instantaneous speeds nm/s	Displacement rate nm/s	Persistency
Chloroplasts (*n* = 10)	29.3 ± 2.0	4.8 ± 0.4	0.17 ± 0.02
Peroxisomes (*n* = 15)	57.7 ± .3.2	7.9 ± 1.4	0.13 ± 0.02
Golgi stacks (*n* = 15)	44.7 ± 2.2	8.3 ± 1.6	0.19 ± 0.04
Mitochondria (*n* = 10)	75.1 ± 3.7	5.9 ± 1.4	0.08 ± 0.02

Number of objects tracked for each organelle is indicated in brackets next to each organelle name.

Values are means ± SE.

## Discussion

### Caulonemata have a specific organelle content different from that of chloronemata

Even though differences in the cytoplasmic organization of moss caulonemata and chloronemata have been noticed decades ago [8,45], previous morphological and ultrastructural studies provided only qualitative information of the sub-cellular content. In this study, we report the first quantitative analysis of organelles distribution in the plant model *Physcomitrella patens*. Using confocal laser scanning microscopy on live cells, we established and validated fluorescent markers for chloroplasts, Golgi dictyosomes, mitochondria and peroxisomes in moss. We showed that caulonemata and chloronemata display a specific organelle content, which is likely to reflect the distinct functions fulfilled by the two cell types. We estimated that caulonemata contain 1.2 to 2.7 times more Golgi dictyosomes than chloronemata depending on the area (figure 5C). The fact that caulonemata grow and divide faster than chloronemata (19.87 ± 2.18 μm h^-1^ vs. 5.85 ± 0.51 μm h^-1^, [7]; 7 h vs. 24 h, [8]) together with our results, may suggest that caulonemata require enhanced trafficking and secretion activity to provide new plasma membrane and cell wall components. Like root hairs in higher plants, caulonemata are thought to be involved in nutrient uptake, a function that necessitates delivery and regulation of several transporters and ATPases at the plasma membrane and is consistent with the central role of the Golgi apparatus in sorting proteins and lipids to different locations at the cell surface [46]. In contrast, we estimated that chloronemata contain 1.7 to 3.7 times more chloroplasts and 1.3 to 6.2 times more peroxisomes than caulonemata depending on the area (figure 5A5B) which is consistent with a high photosynthetic activity. In addition, we observed that peroxisomes are often in close proximity to chloroplasts in both cell types (data not shown). Associations have previously been reported in photosynthetically active palisade mesophyll cells in Arabidopsis [47], and metabolic and physical links between these two organelles have been evidenced in higher plants decades ago [48-50]. Taken together, these data suggest that cross-linked metabolic pathways between chloroplasts and peroxisomes may exist in moss and in the common ancestor with vascular plants. This would be an interesting topic for future investigation.

Interestingly, caulonemata and chloronemata contain a similar density of mitochondria (figure 5D); however, they display very distinct morphology. Consistent with previous reports, morphology and dynamics of mitochondria are known to vary dramatically depending on cell types, tissues, species and physiological conditions. For example, epifluorescent images of Arabidopsis cells expressing a GFP fused to a mitochondrial targeting sequence, showed that hypocotyl cells have spherical mitochondria with uniform diameter (<1 μm) and vascular tissues have sausage-shaped and worm-like structures [51]. It is well accepted that mitochondrial morphodynamics, driven by fusion and fission events, are modulated by bioenergetics, yet the underlying mechanisms remain unclear [52]. Early experiments performed on isolated mitochondria demonstrated that during high energetic level (high ATP concentration), mitochondria are in an "orthodox" state with an intermediate electron-dense expanded matrix and filamentous cristae, while in low energetic level (low ATP concentration), mitochondria are in a "condensed" state with a condensed electron-dense matrix and dilated intercristal spaces [53,54]. These authors hypothesized that the orthodox state was associated with filament-like structures and the condensed state with a fragmented network. Together with our observations, these data support the idea that in moss, caulonemata, which display sausage-like structures, have higher energy requirement than chloronemata, which contain shorter mitochondrial elements. This is in agreement with a study showing that the formation of caulonemata is induced by high energy growth conditions while that of chloronemata is stimulated by low energy growth conditions [55].

### Organelles display an asymmetric distribution in both protonemal filaments but compartmentalization occurs only in tip growing caulonema

In both protonemal filaments, all organelles were distributed in a gradient from the base of the sub-apical cell (zone 1) to the tip of the apical cell (zone 5), the gradient being more dramatic in caulonemata (figure 5). The most important variation we found was for Golgi dictyosomes, which displayed a 9.4 fold difference between the tip of the apical cell and the base of the sub-apical cell in caulonemata (figure 5C). Organelles also exhibit an asymmetric distribution inside a single cell. For example, in tip growing caulonemata, the density of Golgi dictyosomes at the tip (zone 5) was 1.7 and 2.3 times higher than at the nuclear (zone 4) and the basal (zone 3) regions, respectively (figure 5C). One possible explanation for this gradient is that the distal region contains large vacuoles, which are absent or rarely present at the tip [8,9,14], and which could possibly limit the intracellular volume available for other organelles. Furthermore, our quantitative analysis revealed that the first 43 μm of moss caulonemata (zone 5) contains a similar number of mitochondria as the first 40 μm of tobacco pollen tubes, but 10 times more Golgi dictyosomes [21]. One question that arises from these data is why moss apical cells accumulate organelles at their tip? We observed that active chloroplast division occurs in apical caulonemata (see arrowheads in figure 2A and 3A) and only occasionally in sub-apical cells. In addition, it has been well described that the division of peroxisomes is a three-step process that requires sequential elongation, constriction and fission [56]. Peroxisomes which display non-classical filament-like shapes (figure 2A) were detected in apical cells suggesting they could be proliferating and dividing. Similarly, fragmentation of mitochondria was observed at the tip of the apical cells (figure 4A). This is consistent with the fact that division occurs predominantly in apical cells [8] and may suggest that there is asymmetric proliferation and division of organelles, before cell division, to help maintain a constant number of organelles in each daughter cell.

Not only do caulonemata display an asymmetric distribution of their organelles, they also compartmentalize their apical cytoplasm, as chloroplasts and peroxisomes are totally excluded from a 9–15 μm region at the tip. We also showed that there is a 2–3 μm mitochondria-free zone, and, occasionally, a 1–2 μm Golgi deprived-spherical area at the extreme apex (Figures 3 and 4). Based on ultrastructural studies which reported that pollen tubes and root hairs from higher plants, as well as caulonemata from *Funaria hygrometricaPhyscomitrium turbinatum*, dark-grown *Ceratodon purpureus* and *Physcomitrella patens* accumulate vesicles at their tip, we can hypothesize that these organelle-free zones in caulonemata are also packed with vesicles [10-13,20,21,23]. However, there is a high variability in the apical cytoplasmic organization from one type of tip growing cell to another, and there is no clear definition of the clear zone. For example, pollen tubes display a 15 to 25 μm clear zone while those of root hairs and moss caulonema are less than 10 μm ([10-13,20,21,23]; this study). Interestingly, some differences between pollen tubes from different species have also been noticed. For instance, the ER, and some mitochondria and Golgi dictyosomes were occasionally seen in the inverted cone at the extreme apex of pollen tubes from lily, while these organelles were completely excluded from this region in tobacco [20-22]. Together these data raise the question of how this asymmetric distribution is regulated and maintained in tip growing cells, and in particular in caulonemata from *Physcomitrella patens*. In pollen tubes, drug treatments that disrupt the apical F-actin network resulted in inhibition of the delivery of vesicles to the tip of the cells, suggesting that actin filaments present in the clear zone at the apical region could act as a sieve to sort out the secretory vesicles from the bulk of large organelles in pollen tube [17]. Vidali and co-workers reported that tip growing caulonemata from *Physcomitrella patens* also possess an F-actin cortical network at the apical region [57], which seems structurally different from the dense network observed in tobacco pollen tube or from the cortical fringe of short filaments longitudinally oriented in lily pollen tubes, but may fulfill a similar function. For example, all of these structures are highly dynamics and the dynamics of these networks could be responsible for preventing the large organelles from invading the tip of the cells. The fact that chloronemata exhibit a different actin organization that forms a cap underneath the most apical plasma membrane could explain why organelles are not excluded from the tip in these cells.

In addition to the cortical actin, caulonemata from *Physcomitrella* also display a dynamic cluster of F-actin at the extreme apex [57]. It would be interesting to determine if this actin focal point matches the small area deprived of Golgi dictyosomes (see arrow in figure 3) which could also reflect a regulatory mechanism in vesicle trafficking. Furthermore, microtubules could also be involved in maintaining the organelle distribution in *Physcomitrella* as it was shown that treatment with low concentrations (≤ 10^-6^ M) of oryzalin induces tip swelling and migration of plastids into the tip in *Funaria hygrometrica*[58].

In contrast to pollen tubes and root hairs, which display a highly polarized cellular content, thought to be essential for tip growth [15-19], chloronemata exhibit a very slight polarization of their cytoplasm and none of the observed organelles were excluded from their tip, but still expand by tip growth [7]. Therefore, our results support the idea that the compartmentalization of organelles may not be critical for the mechanisms involved in tip growth but may help to achieve higher growth rates.

### Organelles in tip growing caulonemata move slowly and with low persistency

Like in other moss species [9], there is no cytoplasmic streaming in *Physcomitrella.* Several studies have focused on chloroplast movement induced by photorelocation and vacuolar organization in protonemata [14,59,60], but to our knowledge, this work is the first quantitative report on organelle dynamics in *Physcomitrella*. We showed that all organelles tracked move with instantaneous speeds ranging from 29 to 75 nm/s and displacement rates ranging from 4.8 and 8.3 nm/s in caulonemata. Similarly, Pressel et al. (2008) reported that chloroplasts were able to cover distances up to 20 μm in 1 to 6 h in the other moss species *Dicranum scoparium* and *Funaria hyrometrica,* which equals displacement rates of 0.9 to 5.6 nm/s [9]. In contrast, these values are three orders of magnitude slower than those of pollen tube or root hair organelles, for Golgi dictyosomes, mitochondria and peroxisomes were shown to move with speeds in the μm/s range in these tip growing cells [34-37]. Strikingly, there is no correlation between growth rate and cytoplasmic dynamics in tip growing plant cells, as pollen tubes, which grow faster than root hairs, exhibit similar cytoplasmic streaming rates. Another example is that moss caulonemata and root hairs grow at similar rates but display different cytoplasmic dynamics. This is consistent with studies conducted in pollen tubes from lily and tobacco showing that growth and cytoplasmic streaming or secretion can be uncoupled, and that growth strongly depends on a dynamic actin cytoskeleton [61,62].

The analysis of the organelle trajectories also demonstrated that although they move slowly, they can display directionality and low persistency, strongly suggesting that they are most likely not driven by Brownian motion alone, and that the cytoskeleton is likely to be involved in their movements. In tip growing cells from higher plants, drug treatments with latrunculin-B, which affects actin polymerization, and with oryzalin, which depolymerizes microtubules, revealed that ER, vacuoles and mitochondria transport is dictated by the acto-myosin cytoskeleton [22]. Recently, a light-regulated actin-bundling protein was also shown to affect chloroplast movement [63]. Furthermore, YFP fusion proteins with the tail of different myosins revealed that the Arabidopsis proteins MYA and myosinXI-J partially co-localizes with peroxisomes and with Golgi and mitochondria, respectively [64], and that myosin XI-F from Arabidopsis and tobacco interacts with plastids [65]. Expression of tail fragments of six Arabidopsis myosins was found to affect Golgi and mitochondria motility in tobacco [66]. Finally, the recent analyses of knock-out mutants for myosin XI demonstrated that this actin-based motor is critical for the movement of Golgi dictyosomes, mitochondria, ER and peroxisomes in vascular plants [67,68]. In contrast, little is known about the role of microtubules in organelle motility in plants. Kinesins, the microtubule-based motors, were shown to co-localize with Golgi dictyosomes and mitochondria in tobacco pollen tubes [69,70], however, *in vitro* motility assays showed that movements of organelles along microtubule tracks are much slower than cytoplasmic streaming [70,71]. Therefore, microtubules and their motors are not thought to participate in organelle trafficking, but it was proposed that they may finely tune the position of the organelles in the pollen tube [72]. In *Physcomitrella,* there is increasing evidence supporting the idea that organelle transport is mainly regulated by microtubules. First, the genome of *Physcomitrella patens* encodes only three myosin XI homologs compare to 13 isoforms in Arabidopsis [73,74]. Second, myosin XI localization in caulonemata from *Physcomitrella* does not show a specific pattern of accumulation of the organelles analyzed in this study [73], suggesting that myosin XI is not associated with Golgi dictyosomes, mitochondria, chloroplasts or peroxisomes. Third, *in vitro* motility assays performed with purified myosins from lily, tobacco and Arabidopsis showed that these motors move along actin filaments with velocities in the μm/s range [75-78] which is three orders of magnitude higher than the organelle movement in moss. However, the kinesin family of *Physcomitrella* comprises more than 60 members and is comparable to that of seed plants [45]. Furthermore, *in vitro* motility assays showed on one hand that organelles from pollen tubes move along microtubules with velocities ranging from 200 to 300 nm/s [70,71], and on the other hand that kinesins from Arabidopsis and tobacco display sliding velocities along actin filaments between 130 and 400 nm/s which differs only by one order of magnitude from the organelle movement in moss [72,79,80]. Finally, microtubule inhibitor treatments of chloronemal cells of *Physcomitrella patens* and *Funaria hygrometrica* were shown to affect the dynamic organization of vacuoles [14], plastids, ER and mitochondria [9] respectively. One cannot rule out a possible role of the actin cytoskeleton in organelle movement in moss, for it was shown that actin filaments associated with chloroplasts undergo re-organization during chloroplasts photorelocation [60], but the precise mechanism of this motility remains obscure. The purification and characterization of myosins and kinesins from moss cells will be needed to answer the question of what dictates organelle motility in moss.

## Conclusion

In summary, we showed that caulonemata have a specific organelle content different from that of chloronemata and that these specificities are likely to be important for their respective functions. Organelles also display an asymmetric distribution in both protonemal filaments but compartmentalization occurs only in tip growing caulonemata. Further investigation, notably the purification and characterization of molecular motors from moss cells, will help clarify how the organelle organization is maintained and regulated in protonemal cells, and the role of the cytoskeleton. Our data also provide evidence that organelles in tip growing caulonemata move slowly and with lower persistency as compared to those of higher plants.

## Methods

### Culture conditions

All *Physcomitrella patens* lines were propagated using standard methods according to Vidali et al. [6].

For confocal imaging, *Physcomitrella patens* lines were cultured as previously described [73]. Briefly, moss tissues were grown at 25°C under the cycle of 14 h light (90 μmol m^-2^ s^-1^) and 10 h dark for one week on a thin layer of solid PpNO3 medium in glass bottom dishes.

### Generation of organelle marker stable lines

All expression vectors were constructed via multi-site gateway (Invitrogen) using destination vectors kindly provided by Dr Bezanilla and each entry vectors was validated by sequencing.

For the peroxisomal marker, the coding sequence of the CFP, without a stop codon, fused to the peroxisome targeting signal type 1 SKL was amplified from the clone px-ck *CD3-977* (kindly provided by Dr Nebenfür; [30]) with the primers CACCATGGTGAGCAAGGGCGAGG and TTACAGCTTCGATCTCTTGTACAGC, TOPO cloned into the pENTR/D/TOPO vector (Invitrogen) and transferred into the expression vector pTH-Act1Gate via LR reaction.

To create the Golgi marker, the nucleotide sequence encoding the first 49 amino acids of the soybean α-1,2-mannosidase [30,43] was amplified from the clone G-yk *CD3-965* (kindly provided by Dr Nebenfür; [30]) with the primers CACCATGGCTAGCGGGAGCAG and TTACTTGTACAGCTCGTCCATGC, TOPO cloned into the pENTR/D/TOPO vector (Invitrogen) and transfered into the expression vector pTH-35SGate via LR reaction.

The mitochondrial marker was designed by amplification of the coding sequence of the first 29 amino acids of the yeast cytochrome c oxidase IV ScCOX4 [30,44] from the clone mt-rk *CD3-991* (kindly provided by Dr Nebenfür; [30]) using the following primers:

GGGGACAAGTTTGTACAAAAAAGCAGGCTTAATGCTTTCACTACGTCAATCTATAA and GGGGACAACTTTTGTATACAAAGTTGTGGGTTTTTGCTGAAGCAGATATC, and cloned into the donor vector pDONR 221 P1P5r (Invitrogen) by BP reaction. The coding sequence of the mEGFP was cloned into the donor vector pDONR 221 P5P2 (Invitrogen) by BP reaction [81]. The two constructs were inserted into the destination vector pTH-UbiGate via LR reaction.

Protoplast transformation was performed as previously described in Vidali et al. (2007) and all lines were selected on hygromycin-containing medium.

### Confocal imaging

We imaged one week-old moss filaments in which the third cell from the tip had started branching. Five zones, centered either on the tip, the nuclear region or the cell wall region, were defined along the apical and sub-apical protonemal cells expressing a fluorescent organelle marker. Each zone was imaged with a SP5 confocal microscope (Leica) using the 458, 488 and 514 nm argon laser and a dichroic filter to visualize CFP, mEGFP and YFP respectively. The laser power was set at 20% and further attenuated to 20% for the 458 laser and 5% for the 488 and 514 lasers, in order to limit photobleaching and allow the cells to continue to grow during the imaging process. Chloroplasts were tracked based on chlorophyll autofluorescence using the 488 laser at 5%. All images were collected using a PlanApo 40 x oil immersion 1.25 numerical aperture objective. To investigate the distribution of the organelles throughout the cells, between 26 and 74 confocal sections (depending on the volume of the cell), 0.5 μm apart, were acquired for each defined zone and results are displayed as maximal z-projections. To study the mobility of the organelles at the tip of the caulonemata, Z-stacks of 17 slices, 1 μm apart, were collected every 5 s for 5 min and movies (See Additional file 7, Additional file 8, Additional file 9, Additional file 10) are displayed as maximal z-projections.

### Image analysis

All images were processed in ImageJ with an unsharp mask which enhances the edges of the tracked organelles, a smooth function which decreases the graininess, and the pixel values of each slice inside a single Z-stack were normalized using the enhanced contrast function. As the stable lines expressing the mitochondrial marker exhibited a very high background, a deconvolution filter was first used (Iterative Deconvolve 3D plugin in ImageJ). For each zone, the quantification of the organelles inside the Z-stack was performed using the Object Counter 3D plugin in ImageJ (See Additional file 4, Additional file 5, Additional file 6), except for the chloroplasts which were counted manually. To determine the dynamics of organelles, we collected time-lapse series from the stable lines expressing Golgi dictyosomes and peroxisomes fluorescent markers, and the velocity of individual organelles was measured by tracking their center of mass using a semi-automated procedure based on a Gaussian fit of the fluorescence intensity via the SpotTracker2D plugin. The chloroplasts were tracked manually using the threshold function of ImageJ. Contrary to the previous organelles which display predominantly a round morphology, the mitochondria exhibit a filamentous shape and are consequently more challenging to track. Moreover, the population of mitochondria was too dense to use the threshold function. To overcome these technical problems, a Matlab code was written to determine the coordinates of several points assigned to each tracked mitochondrial filament in order to represent its whole shape, and a second code was designed to calculate its corresponding center of mass. These tracking tools allow us to reach a sub-pixel resolution. The speed v is defined by the magnitude of the velocity vector with v = ds/dt where s is the length of the path traveled until time t, and indicates how fast an object moves independently from its direction. The displacement rate was calculated as the shortest traveled distance between the first and the last time points *i.e.* at 5 min interval [82]. The average instantaneous speed representing the limit of the displacement rate as the time interval approaches 0, was calculated as the traveled distance during the smallest interval, *i.e.* at 5 s intervals. The persistency was determined by the ratio average instantaneous speed/displacement rate and describes the straightness of an organelle trajectory. A persistency of 1 indicates that the organelle is moving on a straight line in one direction while a persistency below 1 means that the organelle is changing directions. The persistency was previously referred to as the progressiveness ratio [35] and the meandering index [82]. Results are displayed in Table 1. The trajectories of each tracked organelle are shown in Additional file 11, Additional file 12, Additional file 13, Additional file 14, and two representative trajectories are displayed in figure 6.

## Endnotes

Financial source: WPI-startup funds, National Science Foundation grant (IOS-1002837).

## Authors’ contribution

L.V. and F.F. performed the experimental setup, moss cell line development, data collection, analysis and interpretation. E.T. and K.L. wrote the Matlab routines for data analysis. All authors contributed to drafting the manuscript. All authors read and approved the final manuscript.

## Supplementary Material

Additional file 1 Statistical analysis of organelle densities within filaments. Adjusted P values are shown for rejecting equivalence of means by *t*-test; values in bold indicate that the difference is statistically significant at the 0.05 level. Click here for file

Additional file 2 Statistical analysis of organelle densities within caulonemata. Adjusted P values are shown for rejecting equivalence of means by ANOVA; values in bold indicate that the difference is statistically significant at the 0.05 level. Click here for file

Additional file 3 Statistical analysis of organelle densities within chloronemata. Adjusted P values are shown for rejecting equivalence of means by ANOVA; values in bold indicate that the difference is statistically significant at the 0.05 level. Click here for file

Additional file 4 Peroxisomes quantification in *Physcomitrella patens* protonemata. Fluorescence images and ImageJ-processed images of 5 distinct zones in caulonemata (A) and chloronemata (B) expressing the CFP-SKL fusion protein to quantify peroxisomes (Perox). Images are displayed as maximal projections of confocal sections where each organelle appears under a different color. Scale bar 10 μm. Click here for file

Additional file 5 Golgi dictyosomes quantification in *Physcomitrella patens* protonemata. Fluorescence images and ImageJ-processed images of 5 distinct zones in caulonemata (A) and chloronemata (B) expressing the YFP-Man fusion protein to quantify Golgi dictyosomes (Golgi). Images are displayed as maximal projections of confocal sections where each organelle appears under a different color. Scale bar 10 μm. Click here for file

Additional file 6 Mitochondria quantification in *Physcomitrella patens* protonemata. Fluorescence images and ImageJ-processed images of 5 distinct zones in caulonemata (A) and chloronemata (B) expressing the mEGFP-Cox fusion protein to quantify mitochondria (Mito). Images are displayed as maximal projections of confocal sections where each organelle appears under a different color. Scale bar 10 μm. Click here for file

Additional file 7 Chloroplasts motility in tip growing *Physcomitrella patens* caulonemata. Images were acquired at 5 s intervals for 5 min. Scale bar: 5 μm. Click here for file

Additional file 8 Peroxisomes motility in tip growing *Physcomitrella patens* caulonemata. Images were acquired at 5 s intervals for 5 min. Scale bar: 5 μm. Click here for file

Additional file 9 Golgi dictyosomes motility in tip growing *Physcomitrella patens* caulonemata. Images were acquired at 5 s intervals for 5 min. Scale bar: 5 μm. Click here for file

Additional file 10 Mitochondria motility in tip growing *Physcomitrella patens* caulonemata. Images were acquired at 5 s intervals for 5 min. Scale bar: 5 μm. Click here for file

Additional file 11 Trajectories of chloroplasts in tip growing *Physcomitrella patens* caulonemata. Trajectories have been built from time lapse series in which images were acquired at 5 s intervals for 5 min. Scale unit: μm. Click here for file

Additional file 12 Trajectories of peroxisomes in tip growing *Physcomitrella patens* caulonemata. Trajectories have been built from time lapse series in which images were acquired at 5 s intervals for 5 min. Scale unit: μm. Click here for file

Additional file 13 Trajectories of Golgi dictyosomes in tip growing *Physcomitrella patens* caulonemata. Trajectories have been built from time lapse series in which images were acquired at 5 s intervals for 5 min. Scale unit: μm. Click here for file

Additional file 14 Trajectories of mitochondria in tip growing *Physcomitrella patens* caulonemata. Trajectories have been built from time lapse series in which images were acquired at 5 s intervals for 5 min. Scale unit: μm. Click here for file
