# Supplementary material for: Quantitative analysis of organelle distribution and dynamics in Physcomitrella patens protonemal cells
Source: BMC Plant Biol. 2012 May 17;12:70. doi: 10.1186/1471-2229-12-70 (PMC3476433; doi:10.1186/1471-2229-12-70)
Supplement: Additional file 2 — Statistical analysis of organelle densities within caulonemata. Adjusted P values are shown for rejecting equivalence of means by ANOVA; values in bold indicate that the difference is statistically significant at the 0.05 level. [file 1471-2229-12-70-S2.ppt]

## Slide 1
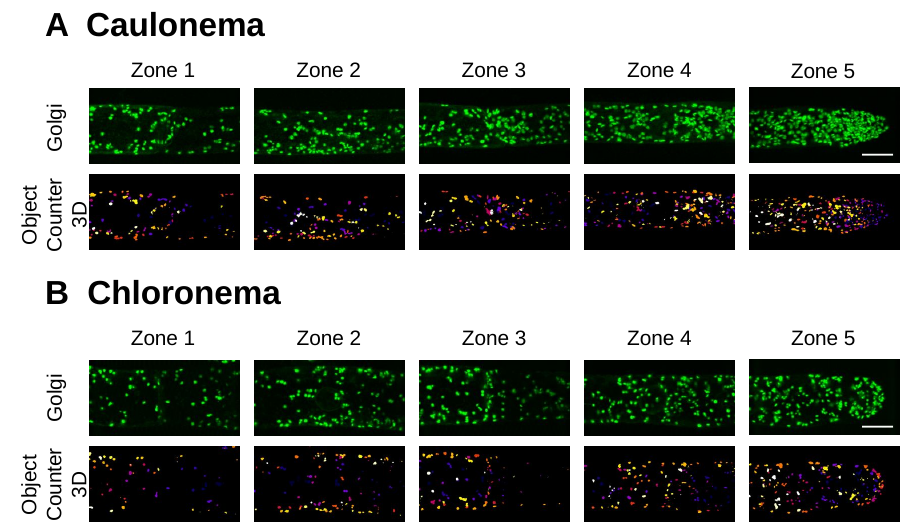

A Caulonema
Zone 1
Zone 2
Zone 3
Zone 4
Zone 5
Golgi
Object Counter 3D
B Chloronema
Zone 1
Zone 2
Zone 3
Zone 4
Zone 5
Golgi
Object Counter 3D
