# Supplementary figures and images for: Quantitative analysis of organelle distribution and dynamics in Physcomitrella patens protonemal cells
Source: BMC Plant Biol. 2012 May 17;12:70. doi: 10.1186/1471-2229-12-70 (PMC3476433; doi:10.1186/1471-2229-12-70)

## Slide 1
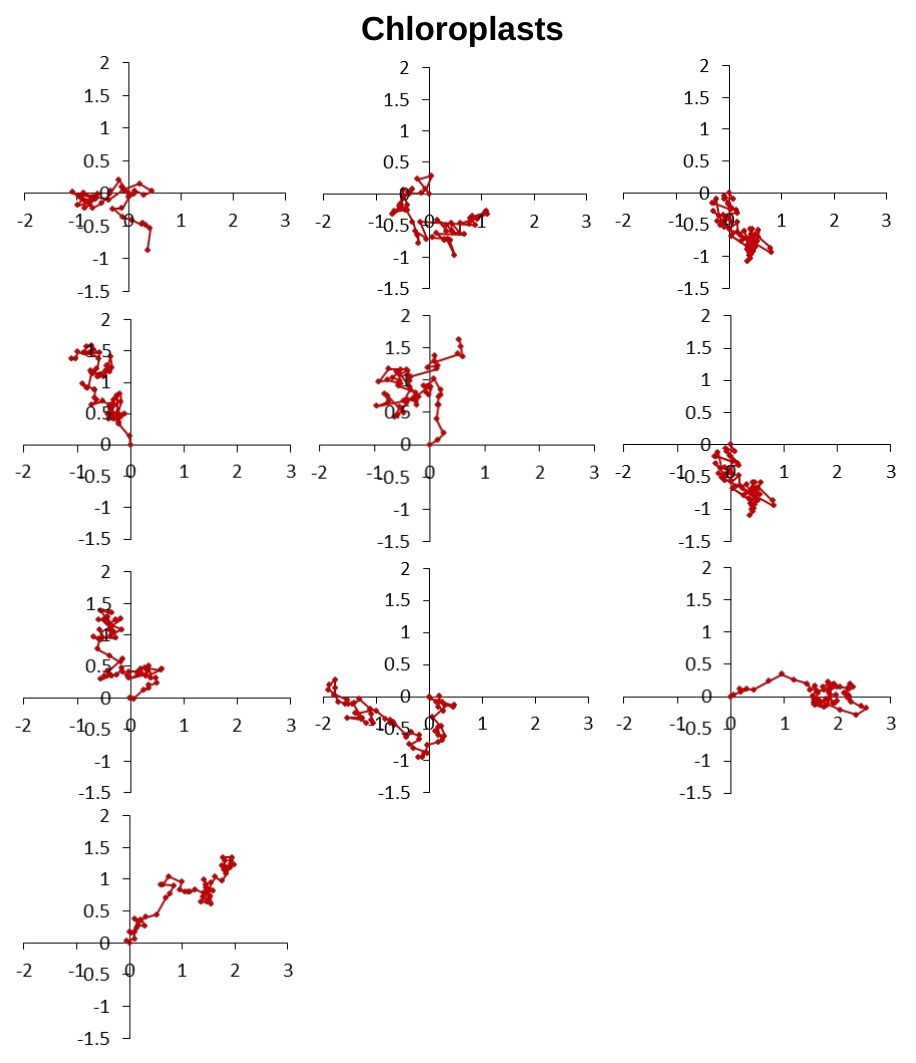

Chloroplasts

Supplement: Additional file 4 — Peroxisomes quantification in Physcomitrella patens protonemata. Fluorescence images and ImageJ-processed images of 5 distinct zones in caulonemata (A) and chloronemata (B) expressing the CFP-SKL fusion protein to quantify peroxisomes (Perox). Images are displayed as maximal projections of confocal sections where each organelle appears under a different color. Scale bar 10 μm. [file 1471-2229-12-70-S4.ppt]

## Slide 1
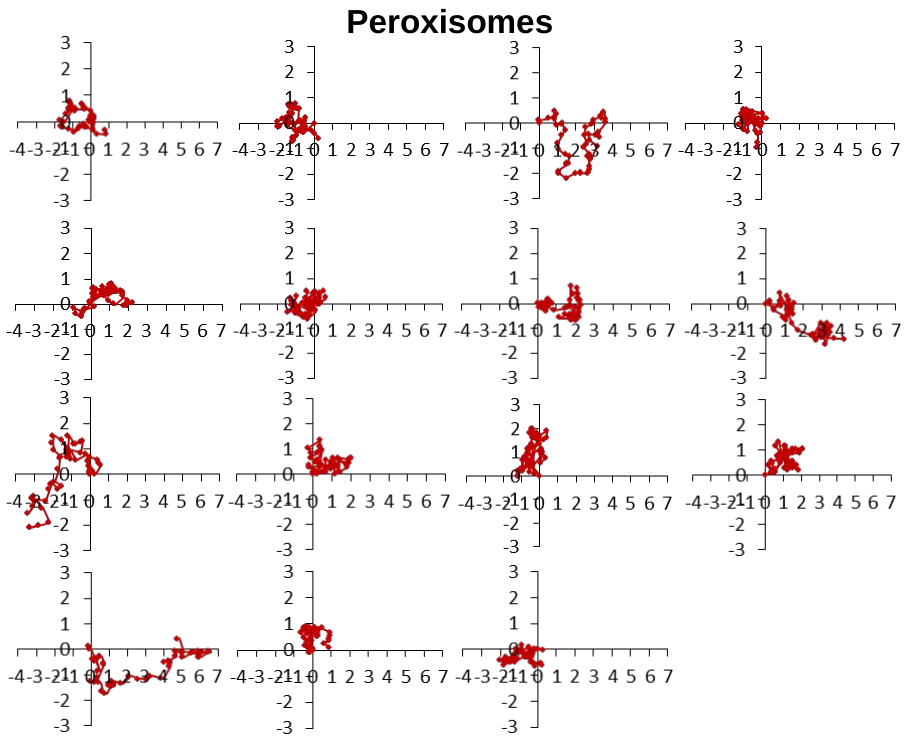

Peroxisomes

Supplement: Additional file 5 — Golgi dictyosomes quantification in Physcomitrella patens protonemata. Fluorescence images and ImageJ-processed images of 5 distinct zones in caulonemata (A) and chloronemata (B) expressing the YFP-Man fusion protein to quantify Golgi dictyosomes (Golgi). Images are displayed as maximal projections of confocal sections where each organelle appears under a different color. Scale bar 10 μm. [file 1471-2229-12-70-S5.ppt]

## Slide 1
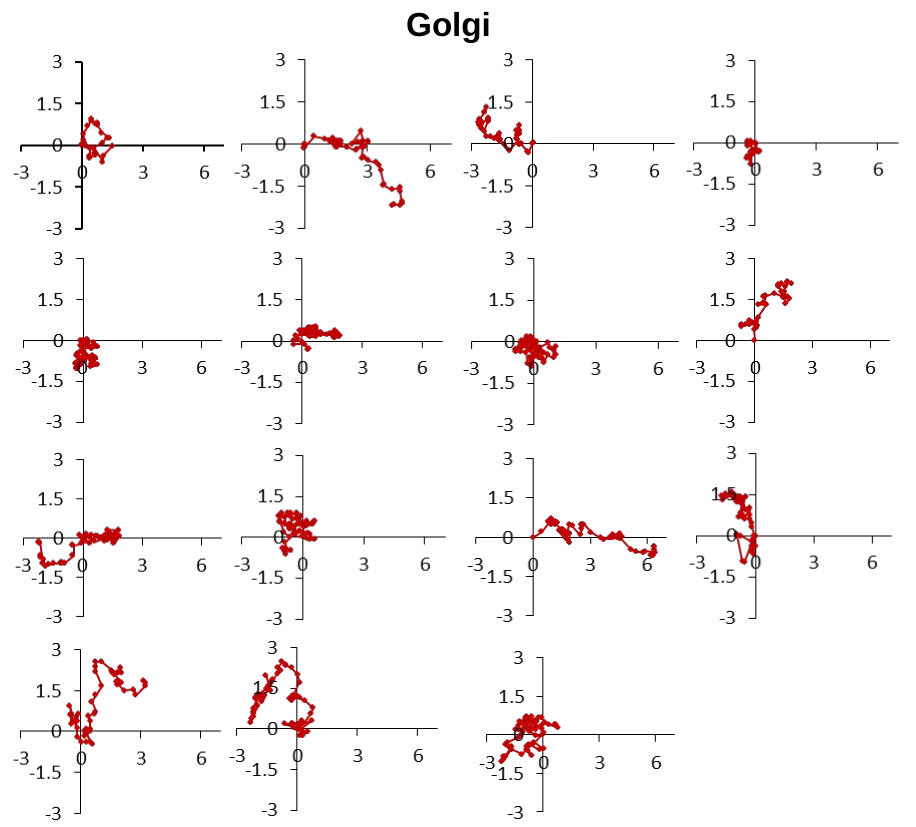

Golgi

Supplement: Additional file 6 — Mitochondria quantification in Physcomitrella patens protonemata. Fluorescence images and ImageJ-processed images of 5 distinct zones in caulonemata (A) and chloronemata (B) expressing the mEGFP-Cox fusion protein to quantify mitochondria (Mito). Images are displayed as maximal projections of confocal sections where each organelle appears under a different color. Scale bar 10 μm. [file 1471-2229-12-70-S6.ppt]

## Slide 1
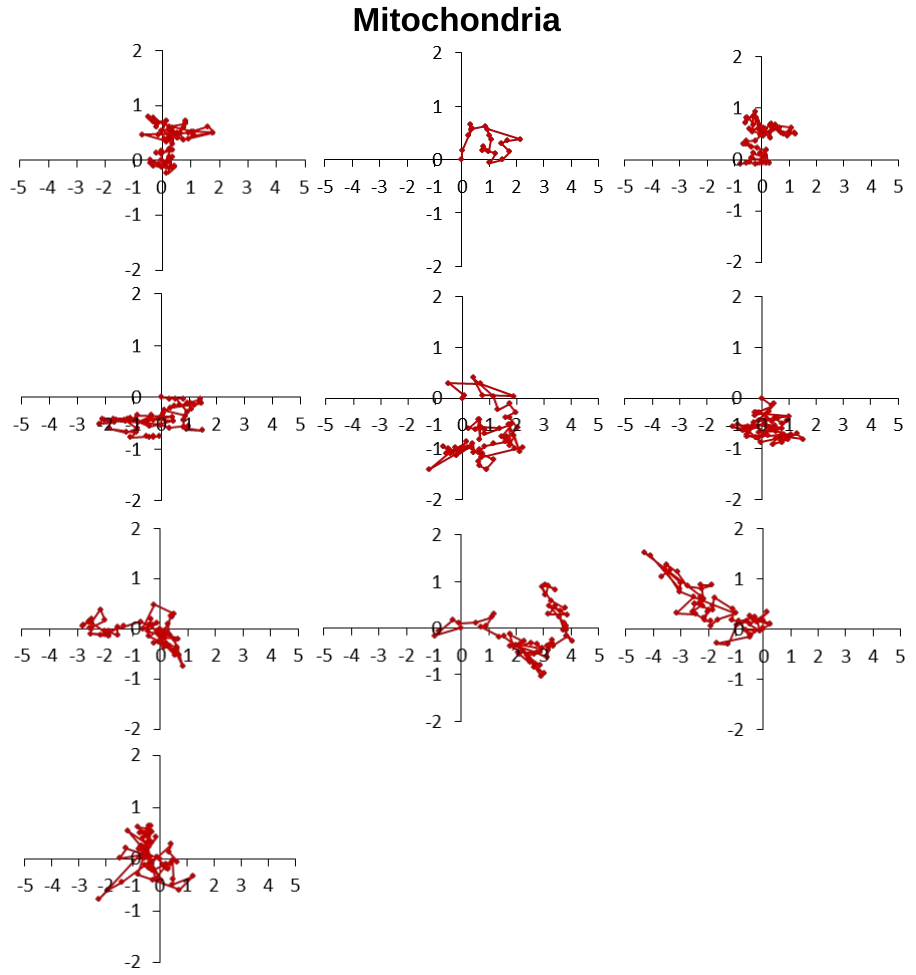

Mitochondria

Supplement: Additional file 7 — Chloroplasts motility in tip growing Physcomitrella patens caulonemata. Images were acquired at 5 s intervals for 5 min. Scale bar: 5 μm. [file 1471-2229-12-70-S7.ppt]
