# Supplementary material for: Quantitative analysis of organelle distribution and dynamics in Physcomitrella patens protonemal cells
Source: BMC Plant Biol. 2012 May 17;12:70. doi: 10.1186/1471-2229-12-70 (PMC3476433; doi:10.1186/1471-2229-12-70)
Supplement: Additional file 8 — Peroxisomes motility in tip growing Physcomitrella patens caulonemata. Images were acquired at 5 s intervals for 5 min. Scale bar: 5 μm. [file 1471-2229-12-70-S8.ppt]

## Slide 1
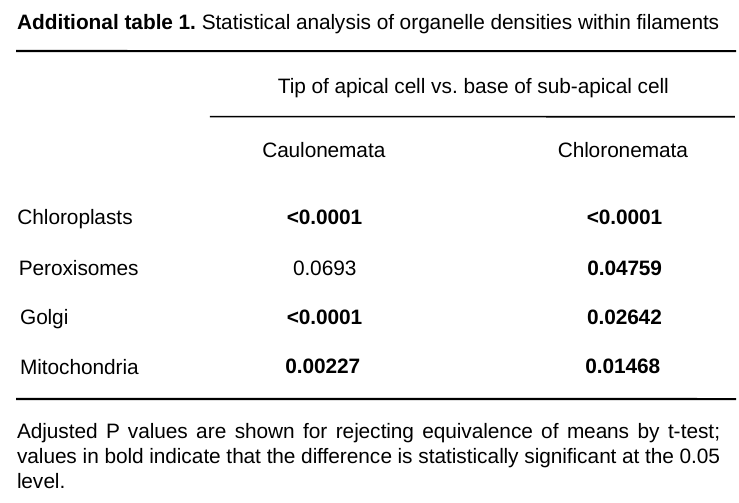

Additional table 1. Statistical analysis of organelle densities within filaments
Tip of apical cell vs. base of sub-apical cell
Caulonemata
Chloronemata
<0.0001
<0.0001
Chloroplasts
Peroxisomes
0.0693
0.04759
Golgi
<0.0001
0.02642
0.00227
0.01468
Mitochondria
Adjusted P values are shown for rejecting equivalence of means by t-test; values in bold indicate that the difference is statistically significant at the 0.05 level.
