# Supplementary material for: Quantitative analysis of organelle distribution and dynamics in Physcomitrella patens protonemal cells
Source: BMC Plant Biol. 2012 May 17;12:70. doi: 10.1186/1471-2229-12-70 (PMC3476433; doi:10.1186/1471-2229-12-70)
Supplement: Additional file 9 — Golgi dictyosomes motility in tip growing Physcomitrella patens caulonemata. Images were acquired at 5 s intervals for 5 min. Scale bar: 5 μm. [file 1471-2229-12-70-S9.ppt]

## Slide 1
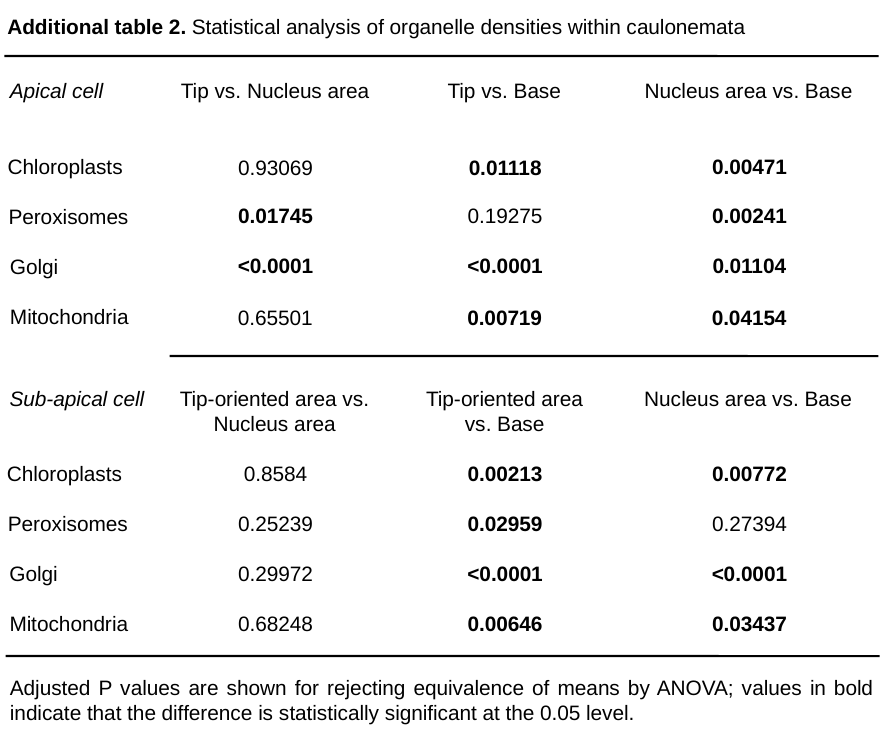

Additional table 2. Statistical analysis of organelle densities within caulonemata
Tip vs. Base
Nucleus area vs. Base
Apical cell
Tip vs. Nucleus area
Chloroplasts
0.00471
0.93069
0.01118
0.00241
0.01745
0.19275
Peroxisomes
0.01104
<0.0001
<0.0001
Golgi
Mitochondria
0.04154
0.65501
0.00719
Tip-oriented area vs. Base
Nucleus area vs. Base
Sub-apical cell
Tip-oriented area vs. Nucleus area
0.00772
0.8584
0.00213
Chloroplasts
0.27394
0.25239
0.02959
Peroxisomes
<0.0001
0.29972
<0.0001
Golgi
0.03437
0.68248
0.00646
Mitochondria
Adjusted P values are shown for rejecting equivalence of means by ANOVA; values in bold indicate that the difference is statistically significant at the 0.05 level.
