# Supplementary material for: Quantitative analysis of organelle distribution and dynamics in Physcomitrella patens protonemal cells
Source: BMC Plant Biol. 2012 May 17;12:70. doi: 10.1186/1471-2229-12-70 (PMC3476433; doi:10.1186/1471-2229-12-70)
Supplement: Additional file 10 — Mitochondria motility in tip growing Physcomitrella patens caulonemata. Images were acquired at 5 s intervals for 5 min. Scale bar: 5 μm. [file 1471-2229-12-70-S10.ppt]

## Slide 1
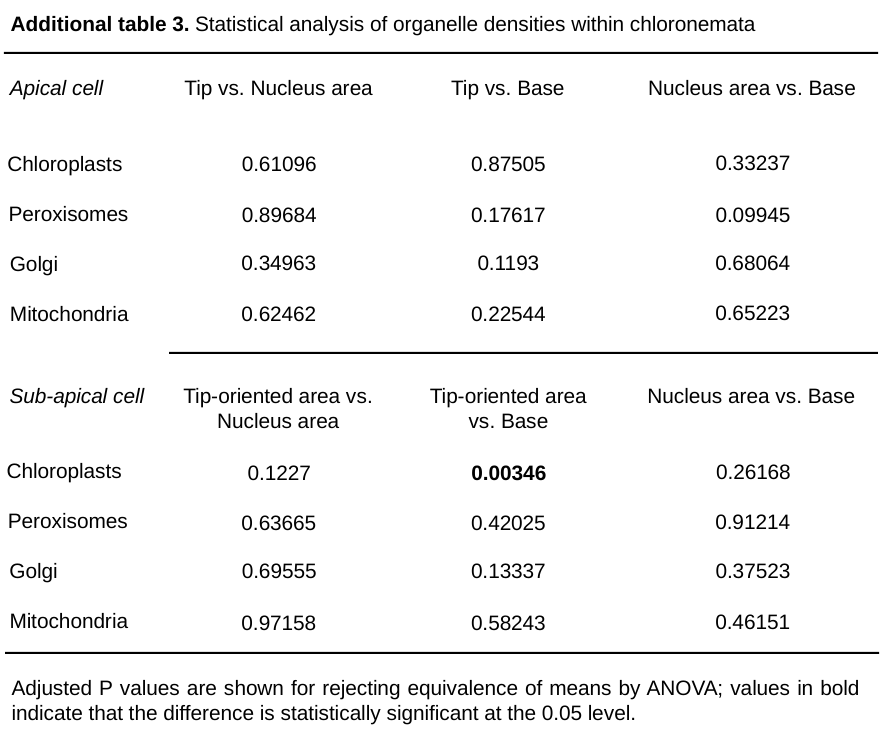

Additional table 3. Statistical analysis of organelle densities within chloronemata
Tip vs. Base
Nucleus area vs. Base
Apical cell
Tip vs. Nucleus area
0.33237
0.61096
0.87505
Chloroplasts
Peroxisomes
0.09945
0.89684
0.17617
0.68064
0.34963
0.1193
Golgi
0.65223
0.62462
0.22544
Mitochondria
Tip-oriented area vs. Base
Nucleus area vs. Base
Sub-apical cell
Tip-oriented area vs. Nucleus area
Chloroplasts
0.26168
0.1227
0.00346
Peroxisomes
0.91214
0.63665
0.42025
0.37523
0.69555
0.13337
Golgi
Mitochondria
0.46151
0.97158
0.58243
Adjusted P values are shown for rejecting equivalence of means by ANOVA; values in bold indicate that the difference is statistically significant at the 0.05 level.
